# Product Roadmap Alignment – Achieving the Vision Together: A Grey Literature Review

**DOI:** 10.1007/978-3-030-58858-8_6

**Published:** 2020-08-18

**Authors:** Stefan Trieflinger, Jürgen Münch, Emre Bogazköy, Patrick Eißler, Jan Schneider, Bastian Roling

**Affiliations:** 6 IT University of Copenhagen, Copenhagen, Denmark; 7 University of British Columbia, Vancouver, BC Canada; 8 Reutlingen University, Alteburgstraße 150, 72762 Reutlingen, Germany; 9 Viastore Software GmbH, Magirusstraße 13, 70469 Stuttgart, Germany

**Keywords:** Product management, Product roadmap, Stakeholder alignment, Business agility, User experience, Objectives and key results

## Abstract

**Context:** A product roadmap is an important tool in product development. It sets the strategic direction in which the product is to be developed to achieve the company’s vision. However, for product roadmaps to be successful, it is essential that all stakeholders agree with the company’s vision and objectives and are aligned and committed to a common product plan. **Objective:** In order to gain a better understanding of product roadmap alignment, this paper aims at identifying measures, activities and techniques in order to align the different stakeholders around the product roadmap. **Method:** We conducted a grey literature review according the guidelines to Garousi et al. **Results:** Several approaches to gain alignment were identified such as defining and communicating clear objectives based on the product vision, conducting cross-functional workshops, shuttle diplomacy, and mission briefing. In addition, our review identified the “Behavioural Change Stairway Model” that suggests five steps to gain alignment by building empathy and a trustful relationship.

## Introduction

An essential aspect for achieving product success in the software-intensive business is that all stakeholders, i.e., all internal and external people who are involved in the product development and related activities (such as engineering, user experience, marketing, sales, suppliers etc.) are aligned and committed around a common product plan. Usually this plan is visualized in the product roadmap. A product roadmap describes how an organization intends to achieve a product vision. It should focus on the value it aims to deliver to its customers and the organization itself in order to rally support and coordinate effort among stakeholders [1]. Consequently, the main purpose of a product roadmap is to provide a high-level view of the direction of the product planning incorporating all key perspectives that supports the strategic dialogue about the future product portfolio. The concept of a product roadmap has changed significantly in recent years. New ways of working such as DevOps, continuous delivery and the increasing use of customer data require to have much more flexible product roadmap formats. Alignment around the product roadmap is important to ensure that each employee is involved in achieving the goals in the product roadmap, so that all activities within product development contribute to achieving the product vision [1]. Alignment means a concerted effort to help people understand the issues and what their respective roles are. Therefore, the product roadmaps will not fulfill its purpose without alignment and buy-in of the key stakeholders. In practice, however, it can be observed that most companies still work in silos, i.e. there is poor communication and cooperation between the different departments of a company [2]. Moreover, stakeholders often do not have consistent and department-specific representations of a common high-level product roadmap that reflects their information needs (e.g., the department product management requires different information on the product roadmap than the department engineering) [3]. One consequence of this situation is that often each department identifies and pursues its own goals and creates its own roadmap independently of the larger goals of the company. Thereby, individual goals are often placed above corporate goals. As a result, not every product activity contributes to achieving the company’s vision and goals, thus wasting important organizational resources [2, 4].

The current scientific literature provides only little knowledge on how alignment can be achieved around a product roadmap [5]. In order to close this gap, the aim of this paper is to identify measures, methods and techniques that help companies to achieve alignment based on the analysis of the so-called “grey literature” (i.e., white papers, articles, blogs, business bocks etc.). It should be stressed that this article refers to alignment of different stakeholders around product roadmaps and not roadmaps in general.

## Related Work

In the scientific literature, few approaches for gaining alignment around a product roadmap can be found. In the following, selected examples are sketched. Khurum and Gorschek [6] describe a method to evaluate the degree of alignment between success-critical stakeholders with respect to the understanding and interpretation of a product strategy. The method also covers misalignments and enables the identification of leading causes. Furthermore, Luftman [7] presents a “strategic alignment maturity assessment tool” which consists of 38 alignment practices grouped into six categories. An organization can evaluate its current maturity level of alignment by giving a score for each alignment practice, averaging the scores for each category and summing up the corresponding average scores. The authors point out that the most valuable part of the assessment is not the assessment itself but understanding its impact on the entire organization and what needs to be done to improve alignment. Barney et al. [8] present a case study in order to understand different levels of alignment between key stakeholders with respect to software quality attributes. As the main reason for the low alignment between different stakeholders, the authors identified among other things insufficiently defined quality requirements and a culture that does not question management decisions. Moreover Lehtola et al. [9] report lessons learned from one software product company that introduced roadmapping processes in order to tie the business viewpoint to requirements engineering decision making and to improve the communication between different stakeholder groups. The authors indicate that if just one person or one function is responsible for the roadmapping process, the other stakeholders may not see the benefits from their viewpoints and therefore feel unmotivated. Finally, Suomalainen et al. [10] point out that typically 1–5 person should participate in the roadmapping process and identified the following as the most important stakeholders of the product roadmapping process: 1) product management, 2) marketing, 3) customer and partner representatives and 4) development including manufacturing and engineering.

## Research Approach

In order to conduct the study in a systematic and repeatable manner, the study at hand follows the guidelines according to Garousi et al. [11]. These guidelines consider three mains phases: 1) planning the review, 2) conducting the review, and 3) reporting the review. The individual phases are described below.

### Planning the Review

#### Identification of the Need of a Grey Literature Review (GLR):

First, we assessed whether a GLR is the appropriate method for our study. For this purpose, we used the checklist developed by Garousi et al. [11]. A recent review of the scientific literature about product roadmaps has shown that most scientific articles do not address product roadmaps operationally or refer to modern product management practices [5]. Furthermore, an initial review of the grey literature on product roadmapping in general and the conduction of expert interviews [4, 12] indicate that the topic product roadmap alignment is highly relevant and of great interest for practitioners. In order to obtain more insights, the conduction of a grey literature review is an appropriate approach and contributes to the transfer of practical knowledge in the scientific community.

#### Formulation of the Research Question:

Based on the study goals we have defined the following research question:

- **RQ1:** Which measures, methods and techniques are reported in the grey literature in order to achieve alignment around the product roadmap?

#### Identification of the Search String:

Our search term was developed in a brainstorming session that aimed at identifying grey literature about product roadmapping in general. In order to obtain sufficient results and cover our objectives we evolved the search term iteratively. At the end of the search process we identified “alignment around the product roadmap” as one of five main issues. Detailed information about the search process can be found in [13]. After evaluating different options, we have defined the following search terms:

A1: Innovation; A2: Product*; A3: Product Management; A4: Agile; A5: Outcome* driven; A6: Outcome* oriented; A7: Goal* oriented; A8: Theme*; A9: Roadmap*

The complete search string used in our study was:

(A1 OR A2 OR A3 OR A4 OR A5 OR A6 OR A7 OR A8) AND A9

#### **Definition of the Inclusion/Exclusion Criteria:**

In order to filter relevant from irrelevant articles, we defined the inclusion and exclusion criteria as shown in Table 1.

**Table 1.** Inclusion and exclusion criteria

Inclusion	• The article discusses the application of product roadmapping in practice
	• The article was published in English
	• The URL is working and freely available
Exclusion	• The source is non text-based
	• The article contains duplicated content of a previously examined article
	• The article is not suitable for software-intensive businesses

### Conducting the Review

#### Conduction of the Study Selection Process.

The data retrieval process was performed by using the predefined search string and applying it to the Google search engine (google.com). In order to avoid biased results based on past activities the search was conducted in the incognito mode of the browser. Further, a VPN service was used to anonymize the location from which the search was conducted. Moreover, the relevance ranking was applied, which ranks the results according to the Google PageRank algorithm. To increase the amount of available URL’s the Google option to include similar results was activated. The search was conducted on January 17^th^, 2020 and yielded in 426 hits. In addition to the search process, we conducted snowballing (i.e., considering further articles that are recommended in an article). This led to 53 further articles. After the application of the selection process (1) scan title, (2) removal of duplicates, (3) applying inclusion and exclusion criteria, (4) scan abstract, (5) scan full text) we obtained 170 relevant articles which address the main topic product roadmapping in a dynamic and uncertain market environment. On this basis we have categorized the 170 articles according to five subject areas: (1) product roadmap formats, (2) product roadmapping processes, (3) product roadmap prioritization techniques, (4) alignment of different stakeholders around the product roadmap, and (5) challenges and pitfalls regarding product roadmapping). This led to 16 relevant articles that deal with the topic alignment of different stakeholders around the product roadmap. Five of these articles are presented in this article. A list of the remaining 11 articles can be found on Figshare [14].

Quality Assessment: The criterion for the quality assessment was that the reviewers were able to comprehend the suggested approach based on their practical experience. In addition, all steps of the selection procedure were carried out individually by two reviewers. In the case that the individual reviews led to different results, the process was carried out by a third reviewer to make a final inclusion/exclusion decision.

## Threats of Validity

We use the framework based on Wohlin et al. [15] as the basis for the discussion of the validity of our study. **Construct validity:** First the construct validity is threatened by the Google search engine regarding the accessibility of search results. After the application of the search string Google returns 78.300.000 articles, but we have only access on 426 articles. We cannot know whether these 426 articles were representative of the total search result of 78.300.000 articles. Moreover, there may be articles that deal with product roadmapping but use the terms that were not covered by our search string. **Internal validity**: In order to mitigate this thread, the quality assessment was conducted by two reviewers independently to limit confirmation bias and interpretation bias. In the case that the individual reviews led to different results, the process was repeated by a third reviewer in order to make a final decision. **External Validity:** The results and conclusion relate to product roadmapping in a dynamic market environment with high uncertainties (e.g., the software-intensive business). Therefore, the results are not directly transferable to other industry sectors. **Conclusion validity**: In order to mitigate this risk, we have presented and discussed our findings with practitioners of the software-intensive business. In this context no major ambiguities or inconsistencies were found [15, 16].

## Results

In order to answer our research question, we analyzed the relevant articles and identified the following measures, methods and techniques that can be used to gain alignment around the product roadmap.

### Foster Alignment with Shared Vision and Goals (OKRs).

Khanna [17] presents an approach to reach vertical and horizontal alignment. Vertical alignment means to make sure that everyone’s goals are aligned across the different layers of a company. In contrast, horizontal alignment represents the collaboration between product teams with other stakeholder such as design, engineering, operation and marketing. In order to achieve a vertical and horizontal alignment, the author recommends as a first step the definition of a clear vision and strategy and its communication throughout the company. Based on the product vision, objectives and key results (OKRs) should be defined, which are broken down and communicated across the different levels of the company. The aim of this activity is that everyone truly understands what strategic direction the company wants to take and how everyone can contribute to the larger goal of the company. In this context, Harke [18] recommends using a mixture of a top-down and bottom-up approach. Besides the definition of a clear vision, Khanna [17] suggests utilizing the following activities: 1) the conduction of weekly progress updates between the management and the product teams in order to ensure that the product activities are focused towards institutional objectives, while fostering transparency across the different levels of the company, 2) the performance of regular cross-functional meetings on the operative level to discuss the future product strategy and eliminate ambiguities regarding the direction of the future product portfolio, and 3) the publication and communication of a product roadmap that lets product teams and stakeholders know which direction the company will take in the future and which topics can be expected.

Lombardo et al. [1] propose shuttle diplomacy, meetings and workshops, and software applications (or some combination thereof) as means to achieve alignment and buy-in around the product roadmap.

### Shuttle Diplomacy.

Shuttle diplomacy involves the conduction of one-on-one meetings with each stakeholder to manage and coordinate their expectations and reach agreement on what the current and future product will be. The idea of this approach is to identify the individual’s goals, priorities as well as other considerations by discussing a draft of the roadmap and reflect whether the stakeholders’ views are in line with the organization’s goals and vision. The one-on-one meetings foster the trust of each stakeholder by listening to them and asking questions why and how things are important for them.

### Meetings and Co-creation Workshops.

The presentation of recommendations at a meeting or the conduction of co-creation workshops can support alignment under certain conditions (e.g., if a culture exists that encourages constructive disagreement). Co-creation workshops are less about presenting a plan and more an interactive event to create a plan. Care should be taken to ensure that the workshops have clearly defined outcomes and a plan for achieving these outcomes before it takes place. A co-creation workshop can follow a shuttle diplomacy effort. However, in some situations (e.g., small teams) it might be useful to skip the one-on-one meetings and go straight to the workshops.

### Software Applications.

Since geographically distributed teams and remote work are becoming increasingly common, it might be difficult to conduct one-on-one shuttle diplomacies or co-creation workshops. Therefore, Lombardo et al. recommend using software applications such as “Roadmunk”, “Aha!” or “ProductPlan”. The combination of such tools with other communications and tracking tools (such as “JIRA”; “Slack”, “GoogleDocs”, “Asana” or “Trello”) can help to create alignment by allowing the teams to agree on topics. The tools also help to raise, manage and track issues and prioritize/reprioritize as necessary.

### Mission Briefing.

Stephen Bungay [19] proposes to create a “mission briefing” as a means to reach alignment. Ideally, the entire product team works out the various sections of the mission letter jointly and iteratively. Since decisions made in one section strongly influence the other sections, it is advisable to work through each section before moving on to the next section. The mission briefing consists of the following five elements: 1) context, 2) higher intent, 3) team intent, 4) key implied tasks, and 5) boundaries. The section “context” describes the current market situation, the problem the product addresses and possible next steps regarding the evolution of the product. The section “higher intent” outlines the overarching corporate strategy and its relationship to specific activities. The identification of customer and business outcomes including appropriate metrics is addressed in the section “team intend”. Within the section “key implied tasks” the forthcoming challenges as well as the persons who act as a contact person for the respective challenges are identified. Finally, the overall scope for the other sections is described in the section “boundaries”.

### Behavioural Change Stairway Model.

Pichler [20] points out that a good way to gain alignment is to carefully listen to the stakeholders, empathise with the stakeholders, and build a trustful relationship. Therefore, the author suggests using the so-called “Behavioural Change Stairway Model” that intends to take the negotiator from listening to influencing the behaviour of other persons. The model consists of the following five stages: 1) active listening (i.e., make an effort to empathically listen to other person while suspending judgement), 2) empathy (i.e., understand the perspective, needs and interests of each individual), 3) rapport (i.e., build rapport and establish trust) 4) influence (i.e., help other persons let go of their position and look for a solution that at least partially addresses the needs of each individual involved), 5) behavioural change (i.e., agree on an acceptable solution).

## Summary

Alignment with a product roadmap is extremely important to ensure that all product development activities contribute to the achievement of corporate goals. The results of the grey literature review presented in this article have in common that achieving alignment should start with a clear vision. Then the vision should be transformed into a strategy with clear goals that can be integrated into a product roadmap and communicated across the organization. This should involve all relevant stakeholders. The aim is that every involved person identifies with this vision and directs his or her activities towards achieving this vision. Besides this, the grey literature review helped to identify several approaches and tactics to gain alignment around the product roadmap. Before applying any of these methods, it should be ensured that the expected results and the purpose of applying the method are clearly defined and communicated to the participants and that the way to achieve the results is well structured.

## References

[1] Lombardo CT, McCarthy B, Ryan E, Conners M (2017). Product Roadmaps Relaunched - How to Set Direction while Embracing Uncertainty.

[2] Lencioni P (2006). Silos, Politics and Turf Wars: A Leadership Fable About Destroying the Barriers that Turn Colleagues into Competitors.

[3] Münch J, Trieflinger S, Lang D, Hyrynsalmi S, Suoranta M, Nguyen-Duc A, Tyrväinen P, Abrahamsson P (2019). The product roadmap maturity model DEEP: validation of a method for assessing the product roadmap capabilities of organizations. Software Business.

[4] Münch J, Trieflinger S, Lang D, Franch X, Männistö T, Martínez-Fernández S (2019). What’s hot in product roadmapping? Key practices and success factors. Product-Focused Software Process Improvement.

[5] Münch J., Trieflinger S., Lang, D.: Product roadmap – from vision to reality: a systematic literature review. In: International Conference on Engineering, Technology and Innovation, ICE/IEEE ITMC. IEEE (2019)

[6] Khurum M, Gorschek T (2011). A method for alignment evaluation of product strategies among stakeholders (MASS) in software intensive product development. J. Soft. Maint. Evol. Res. Pract..

[7] Luftman J (2003). Assessing IT/business alignment. Inf. Syst. Manage..

[8] Barney, S., Wohlin, C., Chatzipetrou, P., Angelis, L.: Offshore insourcing: a case study on software quality alignment. In: Proceedings of IEEE Sixth International Conference on Global Software Engineering, pp. 146–155 (2011)

[9] Lehtola, L., Kauppinen, M., Kujala, S.: Linking the business view to requirements engineering: long-term product planning by roadmapping. In: Proceedings of the 13th IEEE International Conference on Requirements Engineering, pp. 439–443 (2005)

[10] Suomalainen T, Salo O, Abrahamsson P, Similä J (2011). Software product roadmapping in a volatile business environment. J. Syst. Softw..

[11] Garousi V, Felderer M, Mäntylä MV (2019). Guidelines for including grey literature and conducting multivocal literature reviews in software engineering. Inf. Softw. Technol..

[12] Münch J., Trieflinger S., Lang, D.: Why feature-based roadmaps fail in rapidly changing markets: a qualitative survey. In: International Workshop on Software-Intensive Business: Start-ups, Ecosystems and Platforms (SiBW), pp. 202–218. Ceur-WS (2018)

[13] Münch, J., Trieflinger, S., Bogazköy, E., Eißler, P., Roling, B., Schneider, J.: Product roadmap formats for an uncertain future: a grey literature review. Accepted at SEAA (2020). https://bit.ly/prformats. Accessed 30 June 2020

[14] Published on Figshare. https://figshare.com/articles/Product_Roadmap_Alignment_Extended_references/12587759. Accessed 30 June 2020

[15] Wohlin, C., Runeson, P., Hörst, M., Ohlsson, B., Regnell, B., Wesslen, A.: Experimentation in Software Engineering: An Introduction. Kluwer Academic Publishers (2000)

[16] Runeson P, Höst M (2009). Guidelines for conducting and reporting case study research. Empir. Softw. Eng..

[17] Khanna, P.: How to run a product team. https://medium.com/pminsider/how-to-run-a-product-team-fdbee3385c3a. Accessed 1 May 2020

[18] Harke, M.I.: OKR Alignment with OKR examples. https://blog.weekdone.com/okr-alignment-with-examples/. Accessed 1 May 2020

[19] Bungay, S.: The Art of Action: How Leaders Close the Gaps Between Plans, Actions and Results. Nicholas Brealey Publishing, London (2011)

[20] Pichler, R.: How to Lead in Product Management – Practices to Align Stakeholders. Pichler Consulting (2020)

